# Investigation of association of the IL12B and IL23R genes with psoriatic arthritis

**DOI:** 10.1002/art.24128

**Published:** 2008-12

**Authors:** Charlotte Filer, Pauline Ho, Rhodri L Smith, Christopher Griffiths, Helen S Young, Jane Worthington, Ian N Bruce, Anne Barton

**Affiliations:** 1 University of ManchesterManchester, UK; 2 Hope Hospital, and University of ManchesterManchester, UK

## Abstract

**Objective:**

Recent reports have confirmed association of single-nucleotide polymorphisms (SNPs) mapping to the interleukin-23 receptor (IL-23R) and IL-12β genes with psoriasis susceptibility. The aim of this study was to determine whether these variants are also associated with susceptibility to psoriatic arthritis (PsA).

**Methods:**

Two *IL23R* SNPs (rs7530511 and rs11209026) and 2 *IL12B* SNPs (rs3212227 and rs6887695) were genotyped in DNA samples from 520 white patients with PsA and 2,260 control subjects, all of whom resided in the UK. For SNP rs3212227, data on a larger group of controls (n = 4,681) were publicly available; this information was used in the analysis. Genotype counts were compared between patients with PsA and population controls, using the trend test.

**Results:**

A haplotype comprising carriage of the common variants of both *IL23R* SNPs was associated with PsA susceptibility (adjusted *P* = 0.013 [1,000 permutations]). Both *IL12B* SNPs were independently associated with PsA susceptibility, and this association was strongest under a dominant model, with homozygosity for the common allele being more frequent in patients with PsA than in control subjects: for rs3212227, the odds ratio (OR) for carriage of AA versus other genotypes was 1.43 (95% confidence interval [95% CI] 1.17–1.76); for rs6887695, the OR for carriage of GG versus other genotypes was 1.43 (95% CI 1.18–1.74).

**Conclusion:**

Variation within *IL23R* and *IL12B* is associated with susceptibility to both psoriasis and PsA. The effect sizes observed in patients with PsA appear to be smaller than those previously reported in patients with psoriasis, suggesting that both loci are primarily associated with psoriasis susceptibility. However, this does support the idea that the genetic etiology of the psoriasis present in patients with PsA has susceptibility loci in common with those observed in patients with uncomplicated psoriasis.

Psoriatic arthritis (PsA) may be defined as “an inflammatory arthritis associated with psoriasis which is usually negative for rheumatoid factor [RF]” (1). Like other complex diseases, PsA is considered to be triggered by an unknown environmental agent in a genetically susceptible individual. A strong genetic component to disease etiology has been demonstrated, with studies from the UK estimating the sibling recurrence risk (λ_s_) to be 47 and the heritability to be 100% (2). However, few susceptibility genes have been identified, and none have been confirmed as predisposing to PsA independently of psoriasis. For example, several studies have identified association of PsA with the known psoriasis susceptibility allele HLA–Cw∗0602, but the association appears to be primarily with type 1 psoriasis (age at onset of psoriasis ≤40 years) rather than type 2 psoriasis (age at onset of psoriasis >40 years) or PsA itself (3).

Recently, association of single-nucleotide polymorphisms (SNPs) within the interleukin-23 receptor gene (*IL23R*) and a gene encoding a subunit of its ligand, *IL12B*, have been reported to be associated with psoriasis (4). The 2 genes contributed to psoriasis susceptibility independently of each other, and subsequent studies have confirmed association with both genes, which have an additive but not an interactive effect with each other (5,6). Psoriasis and PsA occur together and share the skin phenotype, similarity in cytokine profiles, and the response to anti–tumor necrosis factor biologic therapies, suggesting that they are likely to share some etiologic pathways. Hence, we hypothesized that common genetic factors may underlie these conditions. The aim of the current study was to investigate the role of the *IL23R* and *IL12B* polymorphisms in determining susceptibility to PsA.

## PATIENTS AND METHODS

This study was approved by the North West Multicentre Research Ethics Committee (MREC 99/8/84), and all subjects provided informed consent.

### Samples

DNA samples were available from patients with PsA (n = 520) and population controls. All subjects were white and resided in the UK. PsA was defined as the presence of both psoriasis and inflammatory arthritis, regardless of RF status; details of recruitment to the study have been described previously (3). All cases satisfied the recently published CASPAR (ClASsification criteria for Psoriatic ARthritis) classification criteria for PsA (7). Genotyping was performed on DNA samples obtained from control subjects recruited from among blood donors or from the British 1958 Birth Cohort DNA collection, which is a random subset of individuals born in the UK in one week in March 1958 (n = 2,260). For one SNP (rs3212227), data on a larger group of controls (n = 4,681) were publicly available (http://www.b58cgene.sgul.ac.uk/), and these data were used in the analysis.

### Genotyping

Four SNPs were selected for genotyping. These included the 2 SNPs mapping to the *IL23R* gene, rs11209026 and rs7530511, and the 2 SNPs mapping to the *IL12B* gene, rs6887695 and rs3212227, all of which have previously been associated with psoriasis susceptibility (4–6). Genotyping was performed using the Sequenom MassARRAY platform (Sequenom, San Diego, CA). It should be noted that for 2 SNPs (rs11209026 and rs6887695), the samples from PsA patients have been genotyped previously and the frequencies have been published as part of a separate study (8). Genotype data on patients with type 1 psoriasis presenting to a dermatology clinic in whom a diagnosis of PsA had been excluded (n = 233) were also available, and these data have been published previously as part of a larger cohort of samples from patients with psoriasis (5).

### Statistical analysis

Genotype and allele carriage frequencies were compared between patients with PsA and population controls, using the chi-square test implemented using Stata software (StataCorp, College Station, TX). Haplotype analysis was performed using Haploview software (Broad Institute, Cambridge, MA) with 1,000 permutations, to produce an adjusted *P* value (9).

## RESULTS

Genotype frequencies for both patients with PsA and control subjects conformed to Hardy-Weinberg expectations, and allele frequencies were similar to those reported previously (8).

Both the rs7530511 and the rs11209026 SNPs mapping to *IL23R* showed borderline evidence for association with PsA, using the trend test (Table 1). The rs11209026 SNP encodes an arginine-to-glutamine substitution, and previous reports have suggested that the rare glutamine variant is overrepresented in control subjects compared with patients with psoriasis (10). This was not shown to be the situation for patients with PsA, for whom minor allele frequencies were similar to those in the control population (minor allele frequencies of 0.05 and 0.07, respectively). The *IL23R* SNP rs7530511 also encodes a nonsynonymous substitution of lysine to proline. Although the correlation between the 2 SNPs was low (r^2^ = 0.07), there was evidence for linkage disequilibrium (D′ = 1); therefore, haplotype analysis was undertaken. Carriage of the common haplotype has been previously associated with susceptibility to psoriasis (4). Carriage of the haplotype of common variants was more frequent in patients with PsA than in control subjects (*P* = 0.005). The adjusted *P* value (after 1,000 permutations) remained statistically significant (*P* = 0.013) (Table 2).

**Table 1:** Genotype comparisons between patients with PsA and controls for SNPs mapping to *IL23R* and IL12B*

				OR (95% CI)†		
Locus/gene/genotype	Patients	Controls	*P*, patients versus controls	PsA overall	Type 1 PsA	Type 2 PsA
rs7530511						
*IL23R*						
TT	415 (81.3)	1,663 (77.4)	0.06‡	1.27 (0.99–1.64)	1.35 (1.01–1.81)	1.09 (0.70–1.75)
TC	88 (17.3)	448 (20.9)				
CC	7 (1.4)	36 (1.7)				
rs11209026						
*IL23R*						
GG	469 (90.3)	1,922 (87.0)	0.04‡	1.40 (1.01–1.96)	1.33 (0.93–1.95)	1.59 (0.84–3.31)
GA	48 (9.3)	277 (12.5)				
AA	2 (0.4)	10 (0.5)				
rs3212227						
*IL12B*						
AA	382 (72.5)	3,032 (64.7)§	0.001¶	1.43 (1.17–1.76)	1.32 (1.05–1.68)	1.72 (1.13–2.68)
AC	136 (25.8)	1,478 (31.6)				
CC	9 (1.7)	171 (3.7)				
rs6887695						
*IL12B*						
GG	283 (54.5)	1,021 (45.7)	4.2 × 10^−5^‡	1.43 (1.18–1.74)	1.29 (1.04–1.62)	1.80 (1.23–2.64)
GC	207 (39.9)	1,006 (44.9)				
CC	29 (5.6)	212 (9.4)				

* Values are the number (%). PsA = psoriatic arthritis; SNPs = single-nucleotide polymorphisms; OR = odds ratio; 95% CI = 95% confidence interval.

† Versus other genotypes.

‡ For trend.

§ Information on control genotype frequencies obtained from published data (http://www.b58cgene.sgul.ac.uk/).

¶ Genotypic, calculated using the chi-square test because only summary genotype count data were available.

**Table 2:** Frequency of *IL23R* single-nucleotide polymorphisms in patients with psoriatic arthritis and control subjects*

Haplotype	Patients	Controls	*P*	*P*, adjusted†
TG	85.0	81.2	0.005	0.013
CG	10.1	12.1		
TA	0.05	0.07		

* Values are the percent.

† With 1,000 permutations.

Both of the SNPs mapping to *IL12B*, rs3212227 and rs6887695, showed statistically significant evidence for association (Table 1). For both SNPs, the association was strongest under a dominant model, with 2 copies of the common allele being associated with PsA susceptibility, as has been reported previously with susceptibility to psoriasis (4).

The correlation between the 2 SNPs mapping to *IL12B* was low (r^2^ = 0.21), suggesting that the 2 variants do not lie on a single associated haplotype. Because only summary data, and not individual-level data, were available for control subjects for one of the *IL12B* SNPs (rs3212227), it was not possible to undertake haplotype analysis.

It should be noted that the effect sizes for the 2 SNPs mapping to *IL23R* are lower for PsA than those that have been reported for psoriasis. This may suggest that the primary association is with type 1 psoriasis, because previous reports have been largely restricted to this subgroup. Indeed, in our own series and others, the frequency of HLA–Cw∗0602 is lower in PsA samples than in psoriasis samples, particularly in the type 1 subgroup (3,11). In order to explore this further, patients with PsA were stratified by their age at the onset of psoriasis: patients with type 1 PsA were defined as those who were age 40 years or younger at the onset of psoriasis, and patients with type 2 PsA were defined as those who were older than age 40 years at the onset of psoriasis. Stratification by age at the onset of psoriasis showed that association of the rs7530511 SNP was stronger in patients with type 1 psoriasis. Interestingly, both *IL12B* SNPs showed a trend toward stronger evidence for association in the subgroup of patients with type 2 PsA compared with the control group, although this was based on smaller numbers of patients in the subgroup of patients with type 2 PsA (Table 1).

To further explore whether the primary association is with psoriasis rather than with PsA, carriage of 2 copies of the common allele of each SNP was compared between patients with type 1 psoriasis in whom PsA had been excluded and control subjects. For the *IL23R* SNPs, effect sizes were higher in patients with type 1 psoriasis than in patients with PsA (for rs11209026, OR 2.0; for rs7530511, OR 4.8). For the *IL12B* SNPs, the effect sizes in patients with type 1 PsA were similar to those in patients with type 1 psoriasis (for rs3212227, OR 1.4; for rs6887695, OR 1.3), supporting the hypothesis that the association is primarily with psoriasis.

Subgroup analysis in RF-negative patients with PsA was performed, and the effect sizes in this subgroup were similar to those in the cohort as a whole (OR 1.41, 1.27, 1.45, and 1.49 for rs7530511, rs11209026, rs6887695, and rs3212227, respectively).

## DISCUSSION

In this large case–control study, we observed that polymorphisms in the *IL12B* and *IL23R* genes, both of which were previously associated with psoriasis, are also associated with susceptibility to PsA. The associations of the *IL12B* locus with PsA are stronger than those with the *IL23R* gene (which showed statistically significant evidence for association only when analyzed by haplotype), with carriage of both common variants predisposing to PsA.

The huge overlap in the clinical phenotype of PsA with inflammatory arthritis and psoriasis means that separating the genetic contributions to PsA from the contributions of the constituent components of PsA is a difficult challenge. Because effect sizes reported previously in studies of psoriasis are larger than those observed for PsA in the current study, it is suggested that the association is primarily with psoriasis. Indeed, a recent study of UK patients with psoriasis demonstrated ORs of 1.67, 1.98, 1.38, and 1.72 for the rs11209026 and rs7530511 *IL23R* SNPs and the rs3212227 and rs6887695 *IL12B* SNPs, respectively (5). A subsample of the patients with psoriasis investigated in that study had been specifically examined for the presence of PsA; for those with type 1 psoriasis alone, ORs were higher than or equivalent to the ORs for patients with type 1 PsA, supporting the idea that the primary association is with psoriasis.

Although not all of these polymorphisms have been tested directly for association with inflammatory arthritis, results from imputed data from a large genome-wide association study in 1,860 patients with rheumatoid arthritis (RA) and 2,930 population controls did not show any evidence for association with RA for either the *IL23R* SNPs (for rs11209026, *P* = 0.18 [directly genotyped by the Wellcome Trust Case Control Consortium]; rs7530511, *P* = 0.20 [from imputed data]) or the *IL12B* SNPs (for rs6887695, *P* = 0.40; for rs3212227, *P* = 0.79 [both from imputed data]). Confidence scores for imputed genotypes exceeded 99%, and, at such levels, imputation has previously been shown to be >98.4% accurate (12). Hence, the *IL23R* and *IL12B* loci appear to be associated with psoriasis and PsA but not with inflammatory arthritis in the form of RA.

The possibility that inclusion of RF-positive patients in the current series may have diluted the observed effect of the *IL12B* and *IL23R* variants in PsA susceptibility should be considered, particularly because some of these patients may be found to have RA with coincidental psoriasis. In order to address this possibility, the analysis was repeated, restricted to the RF-negative subgroup. However, effect sizes similar to those seen in the PsA group as a whole were detected, when compared with controls, suggesting that RF is not a significant covariant.

For both psoriasis and PsA, a haplotypic effect at *IL23R*, with carriage of the common variants of both polymorphisms being associated with disease susceptibility, has been observed, suggesting that either both are acting as markers with an as yet ungenotyped variant with which they are both in linkage disequilibrium or that multiple susceptibility effects are present at the same locus. Both *IL23R* SNPs encode nonsynonymous amino acid substitutions. SNP rs11209026 encodes an arginine-to-glutamine substitution at codon 381 (R381Q) within the JAK-2–binding domain, but the results of function studies of this variant have not yet been reported (10).

A haplotypic effect is also seen at the *IL12B* locus, where independent effects of *IL12B* polymorphisms are also present. One of the associated SNPs, rs321227, lies in the 3′-untranslated region of the genes and has previously been reported to affect both IL-12 and IL-12 p40 expression, but findings have been inconsistent. This may be explained by the presence of a second effect within the gene, which was not accounted for in those function studies. Resequencing, fine mapping, and further function studies will be required to explore how these polymorphisms predispose to disease. Interestingly, an anti–IL-12 antibody–based treatment is currently undergoing clinical trials for the treatment of psoriasis, and it will be intriguing to note whether any benefit is also observed in patients with PsA, not only in terms of the skin but also in terms of joint involvement. Indeed, preliminary data (which, to date, have been presented in abstract form only) suggest that a fully human anti–interleukin-12/23 monoclonal antibody might have efficacy in treating inflammatory arthritis in active PsA (13).

In summary, we report association of a haplotype mapping to *IL23R* and 2 SNPs mapping to *IL12B* with susceptibility to PsA. The finding of shared susceptibility between psoriasis and PsA supports the findings of other studies, including studies of the HLA–Cw∗06 locus, which suggest that the genetic factors underlying skin involvement in PsA are no different from those underlying psoriasis without coexistent arthritis. Genome-wide association studies of PsA, therefore, have the potential to identify novel psoriasis susceptibility genes as well as additional PsA susceptibility genes. Results of genome-wide association studies in psoriasis and PsA currently under way will allow comparison with the data from recent RA genome-wide studies and permit a thorough exploration of the overlap between the conditions (14).

## AUTHOR CONTRIBUTIONS

Dr. Barton had full access to all of the data in the study and takes responsibility for the integrity of the data and the accuracy of the data analysis.

**Study design.** Filer, Ho, Worthington, Barton.

**Acquisition of data.** Filer, Smith, Griffiths, Young, Bruce.

**Analysis and interpretation of data.** Filer, Ho, Smith, Griffiths, Young, Worthington, Barton.

**Manuscript preparation.** Filer, Ho, Griffiths, Young, Bruce, Barton.

**Statistical analysis.** Filer, Smith, Barton.
